# Symbiotic Regulatory Genes Controlling Nodule Development in *Pisum sativum* L.

**DOI:** 10.3390/plants9121741

**Published:** 2020-12-09

**Authors:** Viktor E. Tsyganov, Anna V. Tsyganova

**Affiliations:** Laboratory of Molecular and Cellular Biology, All-Russia Research Institute for Agricultural Microbiology, Podbelsky Chaussee 3, Pushkin 8, 196608 Saint Petersburg, Russia; avtsyganova@arriam.ru

**Keywords:** symbiotic genes, nodulation, symbiosis, symbiotic mutants, comparative genetics

## Abstract

Analyses of natural variation and the use of mutagenesis and molecular-biological approaches have revealed 50 symbiotic regulatory genes in pea (*Pisum sativum* L.). Studies of genomic synteny using model legumes, such as *Medicago truncatula* Gaertn. and *Lotus japonicus* (Regel) K. Larsen, have identified the sequences of 15 symbiotic regulatory genes in pea. These genes encode receptor kinases, an ion channel, a calcium/calmodulin-dependent protein kinase, transcription factors, a metal transporter, and an enzyme. This review summarizes and describes mutant alleles, their phenotypic manifestations, and the functions of all identified symbiotic regulatory genes in pea. Some examples of gene interactions are also given. In the review, all mutant alleles in genes with identified sequences are designated and still-unidentified symbiotic regulatory genes of great interest are considered. The identification of these genes will help elucidate additional components involved in infection thread growth, nodule primordium development, bacteroid differentiation and maintenance, and the autoregulation of nodulation. The significance of symbiotic mutants of pea as extremely fruitful genetic models for studying nodule development and for comparative cell biology studies of legume nodules is clearly demonstrated. Finally, it is noted that many more sequences of symbiotic regulatory genes remain to be identified. Transcriptomics approaches and genome-wide sequencing could help address this challenge.

## 1. Introduction

Symbiotic nitrogen fixation has attracted the attention of scientists for more than 100 years. This interest is explained by the exceptional importance of the symbiotic nitrogen fixation process, not only for agricultural production, but also for the fundamentals of biology. The development of a symbiotic nodule is accompanied by various changes in the basic processes that occur in cells. The facultative nature of the formation of nodules makes them a unique model for studying various aspects of the functioning of eukaryotic cells, because it becomes possible to study the manifestations of mutations that disrupt the development of the nodule, which is impossible when studying other vital organs of the plant.

The year 2019 marks the 20th anniversary of the identification of the sequence of the first symbiotic gene *Nodule inception* (*NIN*) in the model legume *Lotus japonicus* (Regel) K. Larsen [1]. A recent comprehensive review summarizes the advances in the identification of symbiotic genes in two model legumes (*L. japonicus* and *Medicago truncatula* Gaertn.) and two crops (*Glycine max* (L.) Merr. and *Phaseolus vulgaris* L.) [2]. However, that review completely ignores advances in the identification of symbiotic genes in pea. This review aims to fill this gap, as well as to show further prospects in this area of research.

Genetic variation in symbiotic traits in peas was discovered as early as the 1920s, when Govorov [3] and later Razumovskaya [4] described local varieties of pea from Afghanistan that were unable to form nodules when inoculated with European strains of rhizobia. In 1964, Gelin and Blixt [5] identified two non-complementary genes in analyses of the ‘Parvus’ pea variety, whose recessive alleles *Pisum sativum nodulation 1* (*Psnod1*) and *Psnod2* determine abundant nodulation; this was a pioneering study in the identification of symbiotic genes in pea.

Since the beginning of the 1980s, experimental mutagenesis has been actively used to discover genetic variation in pea, and has led to the creation of extensive genetic collections [6] and studies [7,8]. Mutagenesis has allowed the detection of regulatory genes present as single copies in the genome. Nonetheless, many legumes have large genomes (for example, in the legume tribe Fabeae, the genome size varies in the range of 1.8–14.3 Gbp/1C [9]), and it is difficult to develop effective transformation protocols for them, which has hampered progress in the identification of important genes. (At the same time, molecular-biological techniques have identified a set of pea genes whose products (nodulins) are significantly increased in nodules [10]; however, these genes are not included in this review, which focuses on regulatory genes.)

In the early 1990s, two legume species began to be actively used as model plants in studies of legume–rhizobial symbiosis: *L. japonicus* and *M. truncatula* [11]. There are several benefits to using such models in molecular-biological studies. For example, they have small genomes, and protocols for effective genetic transformation have been developed for them [12,13]. This has led to the identification of specific sequences of symbiotic genes in model legumes [11]. At the same time, analyses of genomic synteny and microsynteny using model legumes have helped identify symbiotic genes of important crops, including pea [14,15].

More than 50 years have passed since the identification of the first symbiotic genes in pea [5], and amazing progress has been made in obtaining and characterizing pea mutants that exhibit defective nodulation, and determining the sequences of symbiotic regulatory genes and their functions. Nevertheless, many sequences of symbiotic genes remain to be identified. To accelerate the identification of new sequences of such genes, it seems promising to use ‘omics’ technologies.

## 2. Analyses of Natural Variation in the Nodulation Ability of Pea

Since the 1970s, genetic analyses of the primitive pea cultivars ‘Iran’ and ‘Afghanistan’ have identified two symbiotic genes: The dominant *PsSym1* and recessive *Pssym2*. These determine nodulation resistance after pea is inoculated with European rhizobia, but cause the plant to form nodules after inoculation with several rhizobial strains from the Middle East [16,17,18,19]. The presence of the *Pssym2^A^* allele in the cultivar (cv.) ‘Afghanistan’ has been reported [20]. The *PsSym1* allele tends to be dominant and determines temperature-sensitive nodulation [19]. However, *PsSym1* and *Pssym2^A^* are alleles of the same gene [21]. *Pssym2^A^* leads to the interruption of infection thread growth in root hairs when it is initiated with rhizobial strains lacking the *nodX* gene [22]. A study that crossed cv. ‘Trapper’ × cv. ‘Afghanistan’ reported that the *Pssym3* recessive gene in cv. ‘Afghanistan’ led to ineffective nodulation [17]. Other studies have described this same effect caused by the *Pssym6* recessive gene after inoculation with the *Pisum fulvum*-specific *Rhizobium* strain F13 [23,24]. In addition, the *PsSym4* gene has been reported to determine resistance to the *Rhizobium* strain 310a of some genotypes from Afghanistan and Turkey [19]. However, such analyses of natural populations have only allowed the identification of an extremely limited number of symbiotic genes in pea (Table 1).

## 3. Induced Mutagenesis, Identification of Symbiotic Regulatory Genes, and Phenotypic Characterization of Mutants

The advancement of induced mutagenesis has led to the identification of a significant number of symbiotic regulatory genes. Both chemical and physical methods have been used for the mutagenesis of different genotypes: Cv. ‘Rondo’ [39,74,81]; cv. ‘Sparkle’ [28,32]; cv. ‘Finale’ [31]; cv. ‘Frisson’ [36,51]; cv. ‘Bohatýr’ [82]; and lines Sprint-2 [34,55] and SGE [33,49,57] (Table 1).

**Figure 1:**
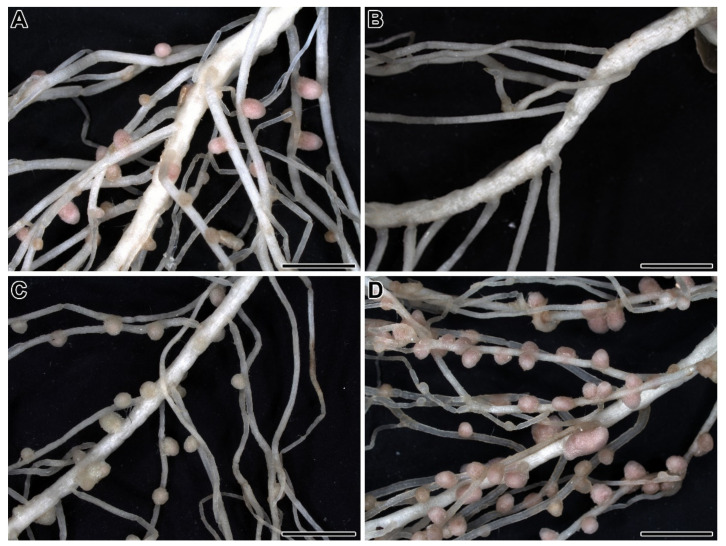
Phenotypes of root systems of wild-type pea and mutants 3 weeks after inoculation with *Rhizobium leguminosarum* bv. *viciae* strain 3841. (**A**) Wild-type Frisson. (**B**) Nod^−^ mutant SGENod^−^-3 (*Pssym35*). (**C**) Fix^−^ mutant SGEFix^−^-3 (*Pssym26*). (**D**) Nod^++^ mutant P64 (*Pssym28*). Bars = 10 mm.

The mutations that have been obtained can be subdivided into four main phenotypic classes (Figure 1) [62]: Unable to form nodules (Nod^−^); forming rare nodules (Nod^+/−^); forming ineffective nodules (Fix^−^); and supernodulating (high number of nodules) (Nod^++^). Mutations belonging to the first two classes make it possible to identify genes that control the early stages of the development of symbiosis; mutations belonging to the third class allow the identification of genes that regulate late stages. The last class includes mutations in genes involved in the autoregulation of nodulation (AON).

It should be noted that some Nod^−^ mutants are also incapable of forming symbioses with endomycorrhizal fungi [83], and common genes involved in the development of both legume–rhizobial and legume–endomycorrhizal symbioses have been identified [14,84]. Such studies have made it possible to make important evolutionary conclusions about the development of legume–rhizobial symbioses based on more ancient endomycorrhizal ones [85].

Complementation analyses of obtained mutants have identified more than 40 symbiotic genes (Table 1). About half of these have been mapped, demonstrating their distribution and linkages (Table 1). Some complementation groups are represented by single mutations and others contain several mutations obtained using different genotypes. 

Many identified mutants have been phenotypically analyzed and studies have identified the different developmental stages of nodules that are interrupted by mutations (reviewed in [7]). The detailed phenotypic characterization of Nod^−^ and Fix^−^ mutants has made it possible to subdivide the genetic program of nodule development into genes that control infection and those involved in nodule morphogenesis [86,87,88]. The process of infection includes the following stages: Root hair curling; rhizobial colonization of the infection pocket (chamber) formed by the curling root; infection thread growth initiation (followed by growth inside the root hair, the root tissue, and then the nodule tissue); infection droplet differentiation; bacteroid differentiation; and nodule persistence. Nodule morphogenesis consists of the initiation of cortical cell division, nodule primordium development, apical nodule meristem development, and nodule meristem persistence [87,88]. 

## 4. Mapping of Symbiotic Regulatory Genes in Pea

Many symbiotic genes in pea have been localized using morphological and molecular markers (Table 1). The largest number of genes are located in the linkage group I, which includes *PsSym2* [25], *PsSym5*, *PsSym10*, *PsSym19/PsSym41* [29], *PsSym33/PsSym11* [59,60], *PsSym35* [15], *PsSym37*, *PsK1* [89], and *PsNod3* [76]. It is interesting to note that all genes encoding the components of Nod factor reception (see below), such as *PsSym2*, *PsSym10*, *PsSym19/PsSym41*, *PsSym37*, and *PsK1*, are mapped to this linkage group. Accordingly, *PsSym33/PsSym11* and *PsSym35* are master regulator genes encoding the key transcription factors involved in Nod factor signal transduction (see below). The *PsNod3* gene encodes a hydroxyproline *O*-arabinosyltransferase (see below). See Table 1 for more details. 

## 5. Identification of Symbiotic Regulatory Genes in Pea

### 5.1. Receptor Kinases

#### 5.1.1. *PsSym10*

Mutants of *PsSym10* were induced in cv. ‘Sparkle’ [32], cv. ‘Frisson’ [36,62], and cv. ‘Finale’ [31] (Table 1). Mutants P5 and P56 are blocked in the very early stages of the pea–*Rhizobium* symbiosis; they lack calcium spiking and root hair deformations in response to Nod factor treatment [62,90]. The *PsSym10* gene is orthologous to *Lotus japonicus NOD FACTOR RECEPTOR KINASE 5* (*LjNFR5*) and *Medicago truncatula NOD FACTOR PERCEPTION* (*MtNFP*) and encodes serine/threonine receptor-like kinase (RLK) with three LysM motifs (LysM) [91,92]. It is interesting to note that in contrast to *LjNFR5*, *PsSYM10* is highly expressed in mature nodules. The mutant alleles for RisFixG, P5, P56, and N16 have been designated *Pssym10-1–4*, respectively. The first three carry nonsense mutations leading to stop codons (after W388, W405, and Q200, accordingly) and result in truncated proteins lacking part of or the entire kinase. *Pssym10-4* is a deletion of the *PsSym10* gene [91]. It is important to note that *PsSym10* is not involved in the control of arbuscular mycorrhizal symbiosis [83]. 

#### 5.1.2. *Pssym37*

Two mutants of *PsSym37* were shown to be induced: K24 in cv. ‘Rondo’ [39] and RisNod4 [31] in cv. ‘Finale’ [89] (Table 1). In K24, the abortion of infection threads in root hairs was observed [39]. The percentage of deformed and curled root hairs in the mutant RisNod4 was twice as high as that of the cv. ‘Finale.’ The growth of the infection thread was usually blocked immediately after its initiation, although some threads were blocked in the root hair and several nodules sometimes formed on the roots of individual plants. Simultaneously, the cortical divisions were activated, and nodule primordia were formed, but not infected [87]. The *PsSym37* gene is orthologous to *LjNFR1* and *MtLYK3* and encodes a LysM-RLK. RisNod4 and K24 have been designated *Pssym37-1* and *Pssym37-2*, respectively. The former carries a missense mutation with a transition of C > T at the 229 position, leading to amino acid substitution in LysM1 domain L77F. A nonsense mutation in K24 presents an allele with a C > T transition, leading to a premature stop codon (after Q539) in the kinase domain [89]. The *PsSym37* gene, like the *PsSym10* gene, is not involved in the control of arbuscular mycorrhizal symbiosis [93].

#### 5.1.3. *PsK1*

The *PsK1* gene was isolated together with the *PsSym37* gene during screening of a cDNA library using LjNFR1 as a probe, and it was also shown to encode a LysM-RLK; PsK1 and PsSYM37 have a high percent of similarity [89]. Three TILLING mutants have been isolated and studied in this gene [73]. The mutants 885, 817, and 2265 have the alleles *Psk1-1*, *Psk1-2*, and *Psk1-3*, respectively. The *Psk1-1* allele carries a mutation leading to an amino acid substitution in the kinase domain (the nucleotide-binding glycine-rich loop)—G332D. The *Psk1-2* and *Psk1-3* alleles carry mutations that result in amino acid replacements in the LysM3 (P169S) and LysM1 (S59F) motifs, respectively. Mutants *Psk1-1* and *Psk1-2* do not form nodules. In *Psk1-1*, rare root hair deformations occur and occasionally, infection threads develop, but they are aborted in the epidermis. In *Psk1-2*, the formation of infection threads is interrupted in the epidermis with the formation of sac-like structures. Nodule primordia are formed, but they are not infected. In *Psk1-3*, part of the infection is also blocked, and nodule formation is delayed [73]. It should be noted that *PsK1* is specifically involved in legume–*Rhizobium* symbiosis, but is not required for the interaction with arbuscular mycorrhizal fungi. However, the increased sensitivity of *Psk1-1* and *Psk1-2* to *Fusarium culmorum* may indicate the possible involvement of PsK1 in the immune response [94]. 

#### 5.1.4. *PsLykX*

*PsLykX* (for *LysM kinase exclusive*) also encodes a LysM-RLK, and was identified by screening the Psa-B-Cam BAC library. It is located near *PsK1* and has a gene structure that is similar to *PsSym37*, consisting of 12 exons and 11 introns. Due to a correlation between the allelic state of *PsLykX* and the specific phenotype of the *Pssym2^A^* allele, this gene was suggested to be the *PsSym2* gene [72]. More recent evidence has emerged to support this identity [95]. During the comprehensive screening of different genotypes from the Middle East, two genotypes from Tajikistan and Turkmenistan were found that had a different allele of *PsLykX*, while in other genotypes, *PsLykX* was found in the allelic state typical for cv. ‘Afghanistan.’ However, all genotypes demonstrated a similar phenotype when the plants were inoculated with European rhizobial strains [95].

#### 5.1.5. Interactions among *PsSYM10*, *PsSYM37*, and *PsK1*

PsSYM10, PsSYM37, and PsK1 may form heteromeric complexes for Nod factor binding, and a model of their interactions has been suggested [73]. According to this model, PsSYM10 and PsK1 form a complex required for the initiation of infection, called a “signaling receptor”. PsSYM10 and PsSYM37, in turn, form a complex involving infection thread progression, called an “entry receptor”. It seems that the entry receptor can also involve PsSYM2 as an additional component. PsK1, together with an as-yet-unidentified co-receptor, may also be involved in the recognition of an unknown signal required for bacterial release [73].

#### 5.1.6. *PsSym19/PsSym41*

The numerous mutants in the gene *PsSym19* were obtained using cv. ‘Frisson’ [36,96], cv. ‘Sparkle’ [28,29], cv. ‘Finale’ [7,31,47], and Sprint-2 [46] (Table 1). The mutants P6 and P55 were blocked in the very early stages of the pea–*Rhizobium* interaction; they lacked calcium spiking and root hair deformations in response to Nod factor treatment [90]. However, the ballooned root hairs in inoculated and uninoculated plants in these mutants have previously been described [62]. The Sprint-2Nod^−^-3 mutant led to the formation of root hairs resembling drumsticks [46]. The *PsSym19* gene is orthologous to *DOES NOT MAKE INFECTION 2* (*MtDMI2*) in *M*. *truncatula* [97] and *SYMBIOSIS RECEPTOR-LIKE KINASE* (*LjSYMRK*) in *L*. *japonicus* [14], which encode leucine-rich repeat receptor kinase. The protein consists of a signal peptide, an extracellular domain (with three leucine-rich repeats), a transmembrane domain, and an intracellular protein kinase domain. The mutant P4 has an allele with a point mutation of G > A in subdomain I of the consensus motif of the glycine-rich loop GXGXXGXV, which is an ATP anchor. The mutant P55 had an allele with a point mutation of G > A in the conserved DFG motif of subdomain VII. Both mutations influence ATP-binding and thus affect catalytic activity [14]. These alleles have been designated *Pssym19-1* and *Pssym19-2*, respectively. The mutant RisFixA has a weak allele containing a 3′-splice-site mutation that influences the proper splicing of intron 9 and leads to a truncated protein lacking the kinase domain [47]. This allele has been designated *Pssym19-3*. In the RisFixA mutant [31], the highly ramified infection thread does not penetrate the nodule primordium, but occasionally, bacterial release occurs, and nodules are formed [88], likely due to the low amount of wild-type PsSYM19 transcript [47]. In these nodules, infection threads are hypertrophied and symbiosomes contain several undifferentiated bacteroids enveloped by the common membrane; these undergo premature degradation [98]. This indicates that PsSYM19 is required for symbiosome development. In the mutants P4 and P55, mycorrhization is completely blocked [83], whereas in RisFixA, mycorrhization developed, although fungal colonization was strongly impaired [47]. Several other mutant alleles of *PsSym19*, such as P6, NEU5, NMU1, RisNod2, RisNod7, RisNod16, RisNod20, and Sprint-2Nod^−^-3, have not been identified. Their identification would be useful for the further elucidation of PsSYM19 functions. 

#### 5.1.7. *PsSym28*

Several mutants in *PsSym28* were induced using cv. ‘Frisson’ [36,53] (Table 1). The mutations lead to supernodulation, shoot fasciation, and the formation of additional flowers. Mutants also demonstrate nitrate-tolerance. The gene encodes a leucine-rich repeat receptor kinase that is similar to AtCLAVATA2 and is involved in AON [53]. The mutants P64, P109, and P113 contain a nonsense mutation (transition G > A) leading to a truncated protein after W456. This allele has been designated *Pssym28-1*. P77, P110, and P120 contain nonsense mutations (transition C > A) leading to truncated proteins after Q618, Q671, and Q638, respectively [53]. These alleles have been designated *Pssym28-2*, *Pssym28-3*, and *Pssym28-4*, respectively. Reciprocal grafting experiments have confirmed that the shoot determines the supernodulation phenotype in *PsSym28* mutants, which indicates that *PsSym28* is expressed in shoots [51,53]. Genes orthologous to *PsSym28* control the perception of CLAVATA3/ENDOSPERM SURROUNDING REGION (CLE) peptides that are transported from root to shoots after the initiation of nodulation and trigger the suppression of further nodulation [99].

#### 5.1.8. *PsSym29*

Numerous mutants in the gene *PsSym29* were obtained using cv. ‘Frisson’ [36] (Table 1). These mutants are also characterized by supernodulation and nitrate-tolerance. The gene is orthologous to *HYPERNODULATION AND ABERRANT ROOT* (*LjHAR1*) and *SUPER NUMERIC NODULES* (*MtSUNN*) and encodes a serine/threonine receptor kinase that is similar to AtCLAVATA1 and is also involved in AON [100,101]. Nine different alleles were identified. The mutant P118 carries a missense mutation (G > A transition) leading to the amino acid substitution V72M in the first leucine-rich repeat. This allele has been designated *Pssym29-1*. Mutants P88, P93, and P119 contain missense mutations (C > A transition) causing the amino acid substitution L290F (allele *Pssym29-2*). P106 also carries a missense mutation (G > A transition) leading to substitution D294N (allele *Pssym29-3*). P122 and P116 contain nonsense mutations leading to truncated proteins (after Q342 and W667, respectively) with complete or partial loss of the kinase domain (alleles *Pssym29-4* and *Pssym29-5*). P90, P91, and P87 carry missense mutations (transitions G > A) leading to amino acid substitutions G695R, G698E, and G831R, respectively (alleles *Pssym29-6* to *Pssym29-8*, respectively). *Pssym29-6* and *Pssym29-7* influence the glycine-rich ATP-binding site of kinase domain I, and *Pssym29-8* affects the kinase activation segment of domain VII. Finally, P89, P94, and P117 carry a nonsense mutation leading to a truncated protein after Q910 (allele *Pssym29-9*) [100]. Reciprocal grafting experiments have shown that the shoot determines the supernodulation phenotype in *PsSym29* mutants, which indicates that *PsSym29* is expressed in shoots [49]. Genes orthologous to *PsSym29* control the perception of CLE peptides [99].

### 5.2. Ion Channels

#### *PsSym8* 

The non-nodulating mutants in the gene *Pssym8* were obtained using cv. ‘Sparkle’ [32], cv. ‘Finale’ [31,102], and Sprint-2 [34] (Table 1). The mutant R25 did not demonstrate root hair deformations upon rhizobial inoculation [103]. However, this mutant, as well as the allelic mutants Sprint-2Nod^−^-1 and Sprint-2Nod^−^-2, showed the abnormal formation of specific spherical swellings of the root hair tips resembling drumsticks [34]. These structures appeared after rhizobial inoculation and their numbers depended on the moisture of the substrate [104]. The mutants RisNod25 and RisNod27 exhibited root hair curling without infection pocket formation in hydroponic solution [105]. Mutants in the gene *PsSym8* lack calcium spikes [90]. The *PsSym8* gene is orthologous to the *M. truncatula DOESN’T MAKE INFECTIONS 1* (*MtDMI1)* gene and *L. japonicus LjPOLLUX* gene [102], which encode the potassium channel and are involved in calcium spiking [106]. The domain structure of PsSYM8 includes transmembrane helices, the filter, the pore helix, the hinge, and the regulation of conductance of the K^+^ (RCK) domain [102]. Five mutant alleles of *PsSym8* have been sequenced. *Pssym8-1* (mutant R25) contains a 1 bp deletion leading to a frame shift and a truncated 229 amino acid peptide. *Pssym8-2* (mutant RisNod10) and *Pssym8-5* (mutant RisNod25) contain missense mutations leading to A306V and R351I substitutions. *Pssym8-3* (mutant RisNod19) and *Pssym8-4* (mutant RisNod21) carry nonsense mutations G2215A and T2834A, leading to stop codons [102]. One more allele was identified in the mutant RisNod27 [107], which was designated *Pssym8-6*. This allele contains a C1676T transition leading to an H309Y substitution. Its suggested role in ion dehydration is in line with observations that the mutant phenotype in RisNod27 can be partially recovered under water stress [107]. Several other mutant alleles of *PsSym8*, Sprint-2Nod^−^-1, Sprint-2Nod^−^-2, E14, R19, R80, and RisNod13 have not been identified. Elucidating these mutants could further clarify PsSYM8 functions. It is interesting to note that mutants R25 and Sprint-2Nod^−^-2 display impaired mycorrhizal development [108,109]. In contrast, RisNod27 exhibits mycorrhizal symbiosis, but decreases mycorrhization [107]. 

### 5.3. Calcium/Calmodulin-Dependent Protein Kinase

#### *PsSym9* 

The non-nodulating mutants in the *PsSym9* gene were obtained using three different genotypes: ‘Sparkle’ [32]; ‘Frisson’ [36,38,62]; and ‘Finale’ [31,35,38] (Table 1). In these mutants, calcium spikes are induced, but root hair deformations are absent [90]; however, the deformations occur upon inoculation with rhizobia in the R72 mutant [103]. The *PsSym9* gene is orthologous to the *DOESN’T MAKE INFECTIONS 3* (*MtDMI3*) gene and encodes calcium/calmodulin-dependent protein kinase (CCaMK) [38]. CCaMK is considered the central component of the Nod factor signal transduction pathway decoding nuclear calcium spikes. LjCYCLOPS is a phosphorylation substrate for the CCaMK [110]. CCaMK consists of a serine/threonine kinase domain, a calmodulin-binding site, calcium-binding EF hand motifs, and an autophosphorylation site. Mutants P1, P2, and P3 have the same allele (*Pssym9-1*), which contains nonsense mutations at the same position leading to a stop codon (after Q230). The alleles *Pssym9-2* (mutant P53) and *Pssym9-3* (mutant R72) carry nonsense mutations leading to stop codons (after W240 and S224, respectively). *Pssym9-4* (RisNod6) contains a 1 bp deletion that leads to a stop codon (after L188). *Pssym9-5* (RisNod9) and *Pssym9-6* (RisNod22) contain missense mutations leading to substitutions G200K and S24F, respectively [38].

### 5.4. Transcription and Co-Transcriptional Factors

#### 5.4.1. *PsSym33*

**Figure 2:**
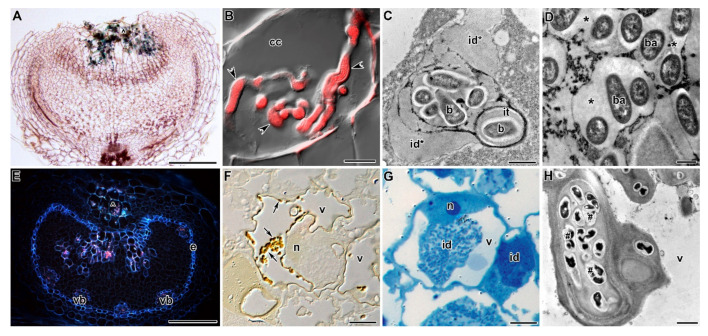
Phenotype of nodules of mutants in the gene *PsSym33* 2 (**H**) and 4 (**A**–**G**) weeks after inoculation with *Rhizobium leguminosarum* bv. *viciae* strains VF39-*gusA* [113] (**A**), 3841 [120] (**B**–**G**), and RCAM1026 [121] (**H**). (**A**) Histological organization of the nodule of mutant SGEFix^−^-2 (*Pssym33-3*); the expression pattern of constitutive *gusA* fusion. (**B**) Highly ramified infection thread in a colonized cell in the nodule of mutant SGEFix^−^-2 (*Pssym33-3*); merging of differential interference contrast (DIC) and the red channel (rhizobia). (**C**) Infection thread with bacteria-free infection droplets in the white nodule of mutant SGEFix^−^-2 (*Pssym33-3*); TEM. (**D**) “Multiple” symbiosomes with undifferentiated bacteroids in the pinkish nodule of mutant SGEFix^−^-2 (*Pssym33-3*); TEM. (**E**) Deposition of suberin in the endoderm and colonized cells in the white nodule of mutant SGEFix^−^-2 (*Pssym33-3*); localization of suberin with Neutral Red. (**F**) Deposition of suberin in the vacuole of the colonized cell in the pinkish nodule of mutant SGEFix^−^-2 (*Pssym33-3*); localization of suberin with iodine and sulphuric acid. (**G**.) Hypertrophied infection droplet in colonized cells of a white nodule of mutant SGEFix^−^-2 (*Pssym33-3*); semi-thin section stained with Methylene Blue-Azur II. (**H**) “Locked” infection threads with clustered degrading bacteria in the root nodule of mutant SGEFix^−^-5 (*Pssym33-2*); TEM. cc—colonized cell, e—endodermis, vb—vascular bundle, ^—cells with infection threads, n—nucleus, v—vacuole, id—infection droplet, id*—bacteria-free infection droplet, it—infection thread, b—bacterium, ba—bacteroid, *—“multiple” symbiosome, and #—clustered bacteria; arrows indicate suberin depositions in the vacuole, and arrowheads indicate ramified infection thread. (**A**) Courtesy of V.A. Voroshilova, and (**B**,**E**) courtesy of K.A. Ivanova. Bars (**A**,**E**) = 200 µm, (**B**,**F**,**G**) = 5 µm, (**C**,**H**) = 1 µm, and (**D**) = 500 nm.

In *PsSym33*, four independent ineffective mutants were obtained: RisFixU (*Pssym33-1*) [31]; SGEFix^−^-5 (*Pssym33-2*) [49]; SGEFix^−^-2 (*Pssym33-3*) [58]; and N24 (*Pssym11* = *Pssym33-4*) [32,60] (Table 1). The *PsSym33* gene is orthologous to the *M*. *truncatula INTERACTING PROTEIN WITH DMI3* (*MtIPD3*) gene and the *L*. *japonicus LjCYCLOPS* gene [111], which encode a key transcription factor involved in nodule development [110,112]. Mutants in this gene manifest a very recognizable phenotype forming white vase-like nodules with a dark pit at the top (Figure 2A) [58]. However, there are some differences between phenotypic manifestations of different alleles. For example, *Pssym33-1* and *Pssym33-2* [113,114] are strong and form only one type of nodule, whereas *Pssym33-4* does not form nodules [60], and *Pssym33-3* is a weak allele that leads to a leaky phenotype, i.e., the formation of two types of nodule (white and pinkish) [58,113]. In white nodules, infection threads are highly ramified (Figure 2A), their walls are thickened (Figure 2B), and there is no bacterial release [58,88]. Infection droplets are occasionally formed, but they do not contain bacteria [115]. However, bacterial release occurs in some white nodules or cells [113]. In pinkish nodules, development is arrested at the stage of bacteroid differentiation (Figure 2D) [58]. The *Pssym33-1* allele contains a mutation at the 5′ splice site (G > A) of intron 3, which impairs the splicing of intron 3 and leads to a stop codon that results in a truncated protein of 390 amino acids. *Pssym33-2* carries a nonsense mutation—C319T—that causes a stop codon at amino acid 107. *Pssym33-3* contains a nonsense mutation that leads to the C1357T substitution, causing a stop codon that results in a truncated protein that lacks the final 60 amino acids that may explain the leaky phenotype [111]. 

Detailed analyses of the *Pssym33-3* allele have revealed that it leads to the activation of strong defense reactions, such as suberin accumulation inside cell walls and infection thread walls (Figure 2E) and the activation of some defense-related genes [116]. Recently, the deposition of newly formed cell wall material was observed around vacuoles and it was accompanied by suberin accumulation (Figure 2F) [117]. In these nodules, the formation of hypertrophied infection droplets was also noted (Figure 2G). In *Pssym33-2* nodules, the strong defense reactions are associated with the clustering of bacteria inside infection threads following their degradation (Figure 2H) [114]. These findings clearly demonstrate that one of the important functions of the *PsSym33* gene is the suppression of defense reactions during nodule development. The *Pssym33-3* mutant also displays impaired mycorrhizal formation and functioning [118,119]. 

#### 5.4.2. *PsSym40*

**Figure 3:**
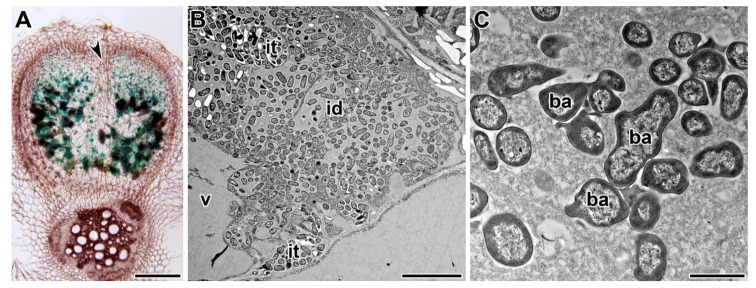
Phenotype of nodules of mutant SGEFix^−^-1 in the gene *PsSym40* after 2 weeks post inoculation with *Rhizobium leguminosarum* bv. *viciae* strains VF39-*gusA* [113] (**A**) and 3841 [120] (**B**,**C**). (**A**) Histological organization of the nodule; expression pattern of constitutive *gusA* fusion. (**B**) Hypertrophied infection droplet in the colonized cell; TEM. (C) Abnormal bacteroids; TEM. v—vacuole, id—infection droplet, it—infection thread, and ba—bacteroid; arrowhead indicates infection thread entry. (**A**) Courtesy of V.A. Voroshilova. Bars (**A**) = 200 µm, (**B**) = 5 µm, and (**C**) = 1 µm.

Two independent alleles of the *PsSym40* gene were obtained after ethyl methanesulfonate (EMS) mutagenesis of the laboratory line SGE: SGEFix^−^-1 (*Pssym40-1*) and SGEFix^−^-6 (*Pssym40-2*) [49,58] (Table 1). Both mutants form numerous small white nodules without histological zonation (Figure 3A) [58,63] as a result of the early halting of the meristem function [88]. *Pssym40-1* leads to the formation of hypertrophied infection droplets (Figure 3B) and abnormal bacteroid development (Figure 3C) [58]. *PsSym40* is orthologous to the *ETHYLENE RESPONSE FACTOR REQUIRED FOR NODULE DIFFERENTIATION* (*MtEFD*) gene [63], which encodes a putative negative regulator of the cytokinin response in nodules [122]. Therefore, it plays a multifunctional role in nodule development, being involved in infection droplet formation and bacteroid differentiation, as well as in control of the nodule number. The *Pssym40-1* mutation leads to the activation of strong defense reactions, such as hydrogen peroxide accumulation around juvenile bacteroids [123,124] and suberization of the nodule endodermis and the vascular endodermis, as well as some defense-related genes [116]. It also displays abnormal mycorrhizal formation and functioning [118,119]. 

#### 5.4.3. *PsSym7*

Four independent non-nodulating mutants in the gene *PsSym7* were obtained using three different pea genotypes [31,32,33] (Table 1). All mutants were unable to form nodules (Nod^−^ phenotype), but they differed in terms of the response of root hairs to rhizobia. The mutant E69 (*Pssym7-1*) induced in cv. ‘Sparkle’ did not exhibit root hair curling [90], whereas RisNod14 (*Pssym7-2*) induced in cv. ‘Finale’ and SGENod^−^-6 (*Pssym7-3*) induced in SGE responded to rhizobial inoculation by forming curled root hairs lacking bacteria [87]. *PsSym7* encodes a GRAS-type transcription factor and is orthologous to the *MtNSP2* and *NODULATION SIGNALING PATHWAY 2* (*LjNSP2*) genes [125,126,127]. The C-terminal domain of PsSYM7 consists of five regions (LHRI, VHIID, LHRII, PFYRE, and SAW), one of which (VHIID) mediates protein–DNA interactions [125]. The *Pssym7-1* allele contains the replacement of an amino acid at position R233, resulting in a premature stop codon in the VHIID region [125]. *Pssym7-2* contains a premature stop codon at the position encoding Q204, which leads to a truncated protein containing the LHRI region only, and *Pssym7-3* contains two missense substitutions (G246E in the VHIID region and M399V in the PFYRE region) [128]. The specific phenotypic manifestation of the *Pssym7-1* allele is probably associated with the altered hormonal status of cv. ‘Sparkle’ compared to cv. ‘Finale’ and the line SGE [128].

#### 5.4.4. *PsSym34*

Three non-nodulating mutants in the *PsSym34* gene (RisNod1, RisNod23, and RisNod30) were obtained using cv. ‘Finale’ [7,31] (Table 1). In RisNod1 and RisNod23, the percentage of deformations and curled root hairs exceeded that in plants of cv. ‘Finale.’ In addition, initiation of the growth of infection threads was delayed, and the number of infection threads was significantly lower. Infection threads grew throughout the root hair, but subsequently, their development stopped in the cells of the outer root cortex [87]. Cortical cell divisions were initiated; however, the process of cell division was stopped, so primordia did not form. The initiation of cortical cell divisions in the root of these mutants was only observed at 23 days after inoculation (DAI), while this occurred at 3 DAI in wild-type plants. Therefore, in mutations in the *Pssym34* gene, the process of nodule tissue development stops at the stage of nodule primordia formation [87]. The *PsSym34* gene encodes the GRAS-type transcription factor and is orthologous to the *NODULATION SIGNALING PATHWAY 1* (*MtNSP1*) gene [129]. MtNSP1 and MtNSP2 form a complex that activates the promoters of genes encoding transcription factors MtNIN and MtERN [130]. Mutations in RisNod1 and RisNod23 have substitutions G1467A and T1296A, respectively, which lead to early stop codons (W489 and C432) and the formation of truncated proteins. These mutations represent two alleles that have been designated *Pssym34-1* and *Pssym34-2*, respectively. The mutations in the *PsSym34* gene influence mycorrhizal development, leading to reduced internal colonization in the early stages of symbiosis development [129].

#### 5.4.5. *PsSym35*

Three non-nodulating mutants in the gene *PsSym35* were obtained using line SGE and cv. ‘Finale’: SGENod^−^-1; SGENod^−^-3; and RisNod8 [31,43,57] (Table 1). All mutants manifested a similar phenotype: The absence of divisions of root cortical cells and a significantly increased number of curled hairs compared to the wild type [43,87]. The recognizable phenotype suggests that *PsSym35* may be orthologous to *NODULE INCEPTION (LjNIN*), representing the first identified symbiotic gene in legumes [15]. The identification of *PsSym35* is an example of the identification of pea genes using genome synteny between crop and model legumes. *LjNIN* encodes a transcription factor with a DNA-binding RWP-RK domain [1]. The MtNIN transcription factor is a key factor coordinating nodule development in different root tissues [131]. The *Pssym35-1* allele (SGENod^−^-1 mutant) has a substitution—C1657T—that creates a stop codon after D552. The *Pssym35-2* allele (SGENod^−^-3) contains the substitution C160T, resulting in a stop codon after P53. *Pssym35-3* (RisNod8) has a substitution—G1210A—causing amino acid substitution E404K, which is embedded in the domain IV [15].

#### 5.4.6. PsKNOTTED1-Related Homeobox3 (*PsKNOX3*)

In pea, inoculation with rhizobia leads to the activation of the *PsKNOX3* gene [132]. At the same time, without inoculation, the overexpression of *PsKNOX3* leads to the formation of nodule-like structures with a central conducting bundle. It has been shown that, in developing nodules, the *PsKNOX3* gene can regulate cytokinin biosynthesis/activation in the nodule [132].

#### 5.4.7. *PsWUSCHEL*-Related Homeobox (*PsWOX5*) 

*PsWOX5* is particularly active in the early stages of nodulation, promoting cell proliferation during the formation of nodule primordia in pea. Furthermore, the suppression of its expression may occur due to autoregulation mechanisms [133].

#### 5.4.8. *PsCochleata (PsCoch)*

The mutants in the gene *PsCoch* have very pronounced phenotypes. Their nodules are able to develop roots at the apical part [134,135]. The gene is orthologous to *NODULE ROOT* (*MtNOOT*) and *Arabidopsis thaliana BLADE-ON-PETIOLE* (*BOP*) and encodes a co-transcriptional regulator involved in the maintenance of nodule identity [71]. 

### 5.5. Transporters

#### *PsSym13* 

Two allelic ineffective mutants in the gene *PsSym13* were obtained using cv. ‘Sparkle’ (E135F and E136) [40] and one was obtained using cv. ‘Frisson’ [41] (Table 1). Detailed analyses of E135F have demonstrated that it blocks nodule development, leading to the formation of ineffective nodules. Bacteroids are morphologically differentiated, but undergo premature degradation [40,55]. The activities of some enzymes involved in carbon and nitrogen metabolism are significantly decreased in the mutant [136,137]. The amount of leghemoglobin is also significantly reduced [138] or even not detected [139]. *PsSym13* is a putative ortholog of the gene *STATIONARY ENDOSYMBIONT NODULE 1* (*LjSEN1*) [42], which encodes a putative Fe transporter in the symbiosome membrane [140]. 

### 5.6. Enzymes

#### *PsNod3* 

Several mutants were identified in the gene *PsNod3* using cv. ‘Rondo’ (nod3 [75], K10a, K11a, and K12a [30]) and cv. ‘Fianle’ (RisFixC [31,77]). These mutants demonstrate supernodulation determined by the root [30,39]. This clearly indicates that PsNOD3 is involved in AON, being involved in the production or transduction of the root-derived signal [141]. *PsNod3* is an ortholog of *ROOT DETERMINED NODULATION1* (*MtRDN1*) [142]. The allele *Psnod3-1* carries a G > A transition mutation, resulting in a stop codon and the synthesis of truncated proteins [142]. PsNOD3 is a hydroxyproline *O*-arabinosyltransferase that post-translationally glycosylates the CLE peptides in the root involved in AON [99].

## 6. Unidentified Pea Symbiotic Genes of Great Interest

### 6.1. PsSym5

Numerous mutants of the gene *Pssym5* were obtained using both EMS and *γ* radiation in cv. ‘Sparkle’ [27] and one more mutant was obtained in cv ‘Ramoskii77’ [30] (Table 1). E2 is the most well-studied mutant. It only forms a few nodules, but their number increases significantly when plants are treated with inhibitors of the action or synthesis of ethylene, as well as when the root systems of mutant plants are cultivated at low temperatures. Mutant plants produce an amount of ethylene similar to the wild type, which indicates an increased sensitivity to ethylene [143]. In the E2 mutant, the abortion of infection threads and premature arrest of cortical cell divisions are observed, which leads to a great decrease in the number of nodule primordia and nodules themselves compared to the wild type [144].

### 6.2. PsSym16

The mutant R50 was induced by exposure to *γ* radiation [28,32] (Table 1). It has a reduced number of nodules compared to the wild type, and shows numerous pleiotropic effects, such as a reduced number of lateral roots; short, thickened internodes and roots; and pale young leaves [145]. It also forms additional vascular poles in primary roots and has an altered vasculature in nodules [146]. Infection threads do not penetrate towards the root stele, but rather branch in enlarged inner cortical cells. Only a few infection threads are associated with cell division and the formation of nodule primordium. Rare primordia are characterized by a flattened shape, being formed by cells that have mainly only undergone anticlinal division. Inhibitors of the synthesis and action of ethylene restored the ability to conduct nodulation in the mutant [145]. At the same time, treatment with exogenous cytokinins of wild-type cv. ‘Sparkle’ plants mimics the mutant phenotype [147]. R50 accumulates elevated amounts of cytokinins in the shoots [148] due to the reduced activity of cytokinin dehydrogenase [149]. Many pleiotropic effects of R50 can be explained by the elevated levels of cytokinins [149]. However, additional pleiotropic effects, such as an increased seed size and the slow emergence of R50 epicotyls, may be determined by abnormal amylase activity and low starch degradation [150]. 

### 6.3. PsSym26

Four independent mutants in the gene *PsSym26* were obtained using cv. ‘Frisson,’ cv. ‘Finale’, and line SGE [31,36,49] (Table 1). The mutants form pinkish ineffective nodules [151], which later change to green [152]. They undergo the premature degradation of symbiotic structures, i.e., manifest early nodule senescence [98,151,153]. Detailed analyses of the SGEFix^−^-3 mutant have revealed that the senescence zone occupies a large part of the nodule in 2-week-old nodules and almost the whole nodule in 4-week-old nodules. Bacteroids demonstrate signs of morphological differentiation in young infected cells, but are degraded in senescence cells, in which remnants of bacteroids and symbiosome membranes are clearly seen [151].

### 6.4. PsSym31

The mutant line Sprint-2Fix^−^ was obtained after EMS-mutagenesis of the laboratory line Sprint-2; it forms white ineffective nodules [34,55] (Table 1). These nodules are characterized by the formation of symbiosomes containing several undifferentiated bacteroids enclosed within one symbiosome membrane [55,154]. Early symbiosome development in *Pssym31* mutants has been confirmed using immunocytological markers [155,156]. For example, the arabinogalactan protein, recognized by the JIM1 antibody, is absent on symbiosome membranes in mutant nodules [156]. The presence of the PsNLEC-1 glycoprotein in the vacuole in mutant nodules instead of symbiosomes indicates the abnormal vesicle targeting pathway implicated in symbiosome development in this mutant [157]. The low level of bacteroid differentiation in Sprint-2Fix^−^ has been confirmed by analyses of colony-forming units from the nodules, which are more abundant than those of wild-type and other mutants [158]. This mutant is characterized by a decreased content of ononitol, altered activity of enzymes involved in nitrogen assimilation, the absence of leghemoglobin [159], and nitrate-tolerance [139]. Therefore, phenotypic manifestations of *Pssym31* mutations appear to constitute a unique, similar phenotype that has not yet been described in other legumes. However, its nucleotide sequence has not yet been sequenced. 

### 6.5. PsSym42

The mutant RixFixV was obtained using cv. Finale [31,45] (Table 1). This mutant forms numerous greenish ineffective nodules with traces of nitrogenase activity [152,160]. A characteristic feature of that mutant is the formation of infection threads with highly enlarged walls [98,153]. Callose depositions are observed in walls of infection threads and host cell walls that have never been observed in other symbiotic mutants of legumes; they, and also degrading bacteroids, have elevated levels of low methyl-esterified homogalacturonan [116].

### 6.6. PsBrz

The mutant E107 was obtained after the EMS mutagenesis of cv. Sparkle [64]. It forms a decreased number of nodules and has, as a pleiotropic effect of mutation, bronze spots on the leaves within 3 weeks after planting. In the mutant, older leaves accumulate 50 times more iron than the wild type [64] and the mutant has higher rates of iron absorption than cv. Sparkle [161]. It also accumulates excessive amounts of aluminum in shoots and roots [162]. 

## 7. Analysis of Types of Interactions among Symbiotic Genes in Pea

The combination of *Pssym12* and *Psnod3* mutations characterized by Nod^−^ and Nod^++^ phenotypes, respectively, in a double recessive homozygote led to a Nod^−^ phenotype, indicating that *Pssym12* is epistatic to *Psnod3*. However, the double mutant forms the compact root system characteristic of the *Psnod3* mutant [39]. Similar results have been obtained for a double recessive homozygote resulting from the crossing of the Nod^++^ mutant K301 and the Nod^−^ mutant K1005M; it does not form nodules, but has fasciated stems, such as those of K301 [80]. At the same time, a double homozygote obtained by crossing the *Pssym37* and *Psnod3* mutants shows less nodulation than the *Psnod3* mutant, indicating that *Pssym37* does not completely epistatically suppress the manifestation of *Psnod3* [39]. Crossing of the supernodulated mutant RisFixC (*Psnod3*) and the ineffective mutant RisFixV (*Pssym42*) produces a double recessive homozygote that forms about 10 times more nodules than cv. ‘Finale.’ At the same time, the parental mutant lines RisFixC (*Psnod3*) and RisFixV (*Pssym42*) form about 5.5 and 4 times more nodules than the wild type, respectively. The additive effect of the combination of these mutants indicates that they are involved in different pathways controlling the nodule number [163].

For Fix^−^ mutants, a set of double-mutant lines has been obtained (RBT, RBT1, RBT3, and RBT4), and the interactions among their genes have been analyzed [115,154,164]. Epistatic interactions of *Pssym31* over *Pssym13*, *Pssym33-3* over *Pssym40-1*, *Pssym40-1* over *Pssym13*, and *Pssym33-3* over *Pssym42* have been observed in terms of nodule histological and ultrastructural organization. The *Pssym33-3* allele is also epistatic over alleles *Pssym40-1* and *Pssym42* in respect to the distribution of low methylesterified pectin labeled with the JIM5 antibody in infection thread walls [116]. *Pssym33-3* and *Pssym31* are epistatic over alleles *Pssym40-1* and *Pssym13*, accordingly, in respect to their influence on the expression of the bacterial genes *nodA* and *dctA* [158] and late bacterial symbiotic genes *fixN*, *fnrN*, and *nifA* [113].

However, some cases of complementary interactions have also been observed. For example, *Pssym31* and *Pssym13* exhibit complementation in respect to the leghemoglobin content [139], and *Pssym33-3* and *Pssym40-1* do so in respect to the abundance and distribution of some epitopes of arabinogalactan protein extensins [165].

A fruitful attempt to use double mutants to elucidate cross-talk between brassinosteroid and strigalacton pathways and the AON pathway has been made. Pea mutants that exhibit the defective biosynthesis of brassinosteroids (*Pslk*) form a reduced number of nodules compared to the wild type [148]. Double mutants for the *Pslk* gene and the *Psnod3*, *Pssym28*, and *Pssym29* genes, which are characterized by impaired AON, display a supernodulating phenotype. These results indicate that brassinosteroids act as positive regulators of nodulation, regardless of the AON system [166]. Pea mutants defective in terms of the biosynthesis of strigalactones of *Psrms1* (*Psccd8*) form a reduced number of nodules [167,168]. In double pea mutants for the *Psrms1* (*Psccd8*) gene and the genes *Psnod3*, *Pssym28*, and *Pssym29*, the supernodulating phenotype was epistatic to reduce the nodule number phenotype. This indicates that strigalactones do not participate in AON, but are involved in the positive regulation of nodulation [166].

## 8. Symbiotic Mutants in Pea as Adequate Genetic Models for Studying Nodule Development and Functioning

### 8.1. Cytokinins and Nodulation

The study of mutant R50 (*Pssym16*) has allowed the identification of the first link between cytokinins and nodule development in pea [145,147]. Another mutant—E151 (*Pssym15*)—which has high levels of cytokinins, has a low nodulation ability, but shows increased mycorrhization, clearly indicating a different role of cytokinins in these two endosymbioses [169]. Analyses of cytokinin responses and the immunolocalization of trans-zeatin riboside and N6-isopentenyladenosine in different symbiotic mutants of pea have revealed coincident abnormalities in nodule development in mutants with abnormal cytokinin responses and localization. These results may indicate that elevated cytokinin levels in the late stages of nodule development may be associated with bacterial release into the host cell cytoplasm and the subsequent differentiation of bacteroids and plant cells [170]. 

### 8.2. Endoplasmic Reticulum Organization

The use of ineffective mutants of pea impaired at different stages of nodule development has made it possible to link the degree of the endoplasmic reticulum network with the level of bacteroid differentiation [171]. For example, in colonized cells in nodules with “locked” infection threads of the mutant SGEFix^−^-2 (*Pssym33-3*) and in infected cells in nodules of the mutant Sprint-2Fix^−^ (*Pssym31*), which contain undifferentiated bacteroids, the endoplasmic reticulum is poorly developed, exhibiting separate segmented tubules beneath the plasmalemma. This pattern corresponds to that of recently infected cells in wild-type nodules. In contrast, in SGEFix^−^-3 (*Pssym26*), which is characterized by morphologically differentiated bacteroids, the endoplasmic reticulum exhibits normal development [171].

### 8.3. Analyses of Nodule Senescence

Nodule senescence is the final stage of its development. Numerous Fix^−^ mutants demonstrate signs of nodule senescence [40,74,98,151,153], and studies have demonstrated a higher activation of nodule senescence triggered by plant mutations, a positive role of ethylene, a negative role of gibberellins, and the universality of nodule senescence as a common plant reaction to nodule ineffectiveness [151,172,173].

### 8.4. Analyses of AON

Grafting experiments on mutants induced in cv. Finale and blocked at different stages of nodule development have clearly demonstrated that the level of nodule development is positively correlated with the degree of AON [174]. In that study, the inoculation of mutants blocked in the earliest stages of nodule development did not significantly inhibit nodulation. The greatest inhibition was observed in the *Pssym32* mutant with the Fix^−^ phenotype. An intermediate level of activation was observed in *Pssym34* (which blocks nodule primordium development and infection thread growth inside root cortex cells) and *Pssym36* (which blocks nodule meristem development and infection thread growth in root hair). These results suggest that AON signaling is correlated with a signal involved in nodule tissue development, but not one involved in infection thread development [174]. 

Recently, a new class of mutants defective in AON has been proposed—the hypo-nodulators [175]. This class includes the *Psbrz* and *Pssym21* mutants, which form fewer nodules than the wild type and mutant phenotypes are shoot-controlled. Additionally, wild-type plants treated with extracts from the shoots of these mutants also display a low nodule number, which suggests the existence of unknown signals involved in AON.

### 8.5. Analyses of Nodulin Gene Expression

A previous study performed comprehensive analyses of the expression of two nodulin genes—*PsENOD12a* and *PsENOD5*—using a large set of symbiotic mutants with 14 defective symbiotic genes, which made it possible to discriminate the functions of the analyzed genes. *PsENOD12a* was found to be involved in early symbiotic stages before infection initiation and *PsENOD5* was found to be required for late symbiotic events after rhizobial penetration into nodule tissues [128]. Differences in the expression levels of several nodulins have also been revealed among Fix^−^ mutants induced on cv Frisson [41].

### 8.6. Analyses of Nitrogen Nutrition and the Yield Relationship

The influence of the nitrogen source (mineral or symbiotrophic) and the level of nitrogen on reproductive development, growth, nitrogen accumulation, and the yield of pea has been analyzed using non-nodulating (Nod^−^), ineffective (Fix^−^), and supernodulating (Nod^++^) mutants, as well as parental cv. ‘Frisson’ grown at different levels of mineral nitrogen under field conditions [176]. The yield of supernodulating mutants was lower than that of the wild type, probably due to the high energy consumption required for nodule development. Only a high dose of mineral nitrogen (50 g N m^−2^) allowed Nod^−^ and Fix^−^ mutants to reach the yield of nitrogen-fixing wild-type plants [41]. This indicates the importance of symbiotrophic nutrition in pea.

### 8.7. Analyses of Nod Factor Induction of Nod Factor Cleaving Enzymes

The use of a large set of pea mutants blocked at the different stages of nodule development has revealed that calcium spiking is required for the activation of lipodisaccharide-forming Nod factor hydrolase, but not for Nod factor-stimulated chitinase [177]. 

### 8.8. Rhizobial Gene Expression

The use of pea mutants blocked in the late nodule developmental stages has demonstrated the gradual downregulation of *nodA* and *dctA*, but not *fixN*, gene expression in rhizobia, which correlates with the degree of bacteroid differentiation [158]. In addition, the expression of the late rhizobial symbiotic genes *fixN*, *fnrN*, and *nifA* requires bacterial release from infection droplets to the host cell cytoplasm, but bacteroid differentiation is not necessary for the induction of these genes [113].

## 9. Comparative Cell Biology

The use of synteny is productive not only for searching and analyzing orthologous genes, but also for analyzing cellular structures involved in symbiosis, for example, the development of infection threads and symbiosomes. Comparative cytological studies have been carried out for pea and *M*. *truncatula*, and both general patterns in the organization of symbiotic and non-symbiotic cell components, and species-specific features, have been identified [156,178]. In the nodules of pea and *M*. *truncatula*, cortical microtubules and endoplasmic microtubules associated with infection thread growth, infection droplet formation, and bacterial release into the host cell cytoplasm show similar patterns, whereas there is a strong difference in the patterns of endoplasmic microtubules around symbiosomes [178].

**Figure 4:**
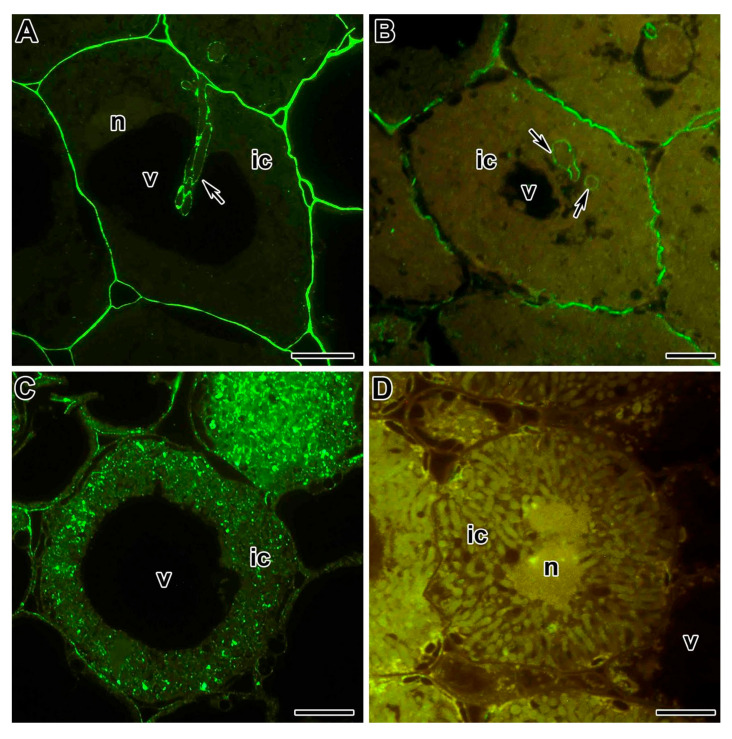
Fluorescent immunolocalization of different epitops of cell wall and plasma membrane components in the wild-type nodules of *P. sativum* and *Medicago truncatula*. The secondary antibody used was goat anti-rat IgG MAb conjugated with Alexa Fluor 488. (**A**) Highly methylesterified homogalacturonan epitope labeled with JIM7 in the nodule from the wild-type line SGE of *P. sativum*. (**B**) Highly methylesterified homogalacturonan epitope labeled with JIM7 in the nodule from wild-type line A17 of *M. truncatula*. (**C**) Arabinogalactan protein epitope labeled with JIM1 in the nodule from wild-type line SGE of *P. sativum*. (**D**) Arabinogalactan protein epitope labeled with JIM1 in the nodule from wild-type line A17 of *M. truncatula*. ic—infected cell, n—nucleus, and v—vacuole; arrows indicate infection threads. Bars = 5 µm.

JIM5 and JIM7 antibodies, which recognize low and highly methylesterified pectins, respectively, show similar labeling patterns in pea and *M*. *truncatula* nodules (Figure 4A,B). At the same time, the LM5 antibody, which recognizes the galactan side chain of rhamnogalacturonan I, labeled infection thread walls in pea nodules, but did not label them in *M*. *truncatula* nodules [156]. A membrane-anchored arabinogalactan protein recognized by JIM1 is present in the plasma and symbiosome membranes of pea nodules, but only in the plasma membranes of *M*. *truncatula* nodules (Figure 4C,D) [156].

Species-specific differences can be explained by the significant difference in bacteroid morphology between analyzed species. Therefore, research in the field of comparative cell biology of model and agricultural legumes is necessary to identify the general mechanisms of the development of legume–rhizobial symbiosis. At the same time, it is important to study individual species to identify the intraspecific features of the formation of nitrogen-fixing nodules.

## 10. Use of Non-Symbiotic Mutants to Study Nodulation

Mutants with altered levels of phytohormones or an altered sensitivity to them have proven to be useful models for revealing the role of phytohormones in nodulation. The level of gibberellins is reduced in mutants of the *Psls-1* and *Psna-1* genes, which is accompanied by the formation of a reduced number of aberrant nodules [148,179], while adding exogenous gibberellins increases the number of mutant nodules. In contrast, the *Pslh-2* mutant is characterized by an increased level of gibberellins, which leads to a decrease in the number of nodules. These data indicate the need to maintain a certain optimal level of gibberellins for the development of symbiotic nodules [148].

The other interesting model for studying nodulation is the mutant SGEcrt (*Pscrt*), which forms short, curly roots in a high-density substrate (e.g., quartz sand), but a normal root system in a low-density substrate (e.g., vermiculite) [180]. The mutant is characterized by increased IAA production [180], as well as elevated ethylene levels and sensitivity [181], and forms fewer nodules than the wild type [182].

Cadmium is a very toxic and dangerous element for plants and symbiotic systems. That is why the identification of molecular-genetic mechanisms of plant tolerance to cadmium is of great importance [183]. The mutant SGECd^t^ is characterized by an increased tolerance to cadmium and elevated cadmium accumulation [184]. The mutant demonstrates a higher tolerance to cadmium, both in terms of nodulation [185,186] and the functioning of established nodules [187].

## 11. Conclusions and Future Perspectives

The study of the genetic control of nodulation in pea has come a long way from the discovery of natural variability in nodulation traits to the identification of nucleotide sequences of symbiotic genes. It should be noted that symbiotic mutants remain the favorite genetic models that are actively used to analyze various aspects of nodule development. However, two-thirds of the symbiotic genes in pea remain unidentified. Therefore, the efforts of researchers in different laboratories around the world should be consolidated to accelerate progress in the identification of symbiotic genes in pea. It seems interesting to elucidate the possible existence of species-specific symbiotic genes, the presence of which may be associated, for example, with species-specific forms of bacteroids. 

The development of ‘omics’ technologies could be extremely useful for accelerating the identification of symbiotic regulatory genes in pea. To date, only a few studies of the transcriptomes of pea nodules of cv. ‘Caméor’ [188] and the line SGE [189] have been performed. Analyses of the transcriptomes of nodules of cv. ‘Caméor’ have allowed the identification of candidate genes for pea orthologs of the main symbiotic regulatory genes in *M. truncatula* and *L. japonicus.* Many of these genes have been found to have similar spatiotemporal expression patterns as genes in *M. truncatula* nodules [188]. However, further characterization of candidate genes is required to elucidate their function in nodule development in pea. Indeed, the detailed study of transcripts identified in nodule transcriptomes in the line SGE has revealed that the *PsSym34* gene is a potential homolog to the *MtNSP1* gene [129]. 

Currently, three pea genome assemblies are available: Those for cv. ‘Caméor’ [190]; cv. ‘Gradus No 2’) [191]; and line JI 128 [192]. These could represent another useful tool for identifying symbiotic regulatory genes in pea. 

Finally, it is necessary to note that understanding the genetic control of nodulation in pea should lead to strategies to significantly help increase the efficiency of nitrogen fixation and make the use of peas in sustainable agriculture more attractive.

## Figures and Tables

**Table 1:** Symbiotic genes of pea (*Pisum sativum* L.).

Symbiotic Locus *	Linkage Group	Phenotypes	Mutant Lines	References
*Sym1 = sym2*	I	Nod^+/−^	JI 1357 (registered type line), VIR K320-1	[16,17,20,21,25,26]
*sym3*	-	Fix^−^	JI 1357 (registered type line)	[17]
*Sym4*	-	Nod^−^	JI 261	[19]
*sym5*	I	Nod^−^	E2, R88, E77, E111, E143, E166, E169, K20a	[27,28,29,30]
*sym6*	-	Fix^−^	JI 1357 (registered type line)	[23,24]
*sym7*	III	Nod^−^	E69, N12, RisNod14, SGENod^−^-2	[7,31,32,33]
*sym8 = sym20*	VI	Nod^−^	E14, R19, R25, R80, RisNod10, RisNod13, RisNod19, RisNod21, RisNod25, Sprint-2Nod^−^-1, Sprint-2Nod^−^-2	[7,31,32,34,35]
*sym9 = sym30*	IV	Nod^−^	R72, P54, P1, P2, P3, P53, RisNod6, RisNod9, RisNod22	[7,8,31,32,35,36,37,38]
*sym10*	I	Nod^−^	P5, P7, P8, P9, P10, P56, RisFixG	[7,31,32,36]
*sym12*		Nod^+/−^	K5	[39]
*sym13*	VII	Fix^−^	E135f, E136, P58	[40,41,42]
*sym14*	III	Nod^−^	E135n, SGENod^−^-2	[29,40,43]
*sym15*	VII	Nod^+/−^	E151	[32]
*sym16*	V	Nod^−^	R50	[32]
*sym17*	VI	Nod^+/−^	R82	[32]
*sym18*	II	Nod^+/−^	E54	[29,44]
*sym19 = sym41*	I	Nod^−^/Fix^−^	P4, P6, P55, NEU5, NMU1, RisNod2, RisNod7, RisNod16, RisNod20, Sprint-2Nod^−^-3, RisFixA	[7,28,31,36,45,46,47]
*sym21*	-	Nod^+/−^	E132	[48]
*Sym22*	II	Nod^+/−^	JI 1794	[10]
*sym23*	-	Fix^−^	P59	[7,36,41]
*sym24*	-	Fix^−^	P60	[7,36]
*sym25*	-	Fix^−^	P14, P17, P19, P61, SGEFix^−^-8	[7,36,49]
*sym26*	-	Fix^−^	P63, RisFixM, RisFixT, SGEFix^−^-3	[7,31,33,36]
*sym27*	V	Fix^−^	P12, RisFixQ, SGEFix^−^-7,	[7,31,36,42,49,50]
*sym28*	V	Nod^++^	P64, P77, P109. P110, P113, P120	[51,52,53]
*sym29*	VII	Nod^++^	P87, P88, P89, P90, P91, P93, P94	[51,54]
*sym31*	III	Fix^−^	Sprint-2Fix^−^	[55,56]
*sym32*	-	Fix^−^	RisFixL, RisFixO	[7,31]
*sym33 = sym11*	I	Fix^−^/Nod^−^	RisFixU, SGEFix^−^-2, SGEFix^−^-5, N24	[7,31,32,49,57,58,59,60]
*sym34*	-	Nod^−^	RisNod1, RisNod23, RisNod30	[7,31]
*sym35*	I	Nod^−^	RisNod8, SGENod^−^-1, SGENod^−^-3	[15,31,43]
*sym36*	-	Nod^−^	RisNod24, RisNod26	[7,31]
*sym37*	-	Nod^+/−^	RisNod4, K24	[7,31,39]
*sym38*	V	Nod^−^	RisFixF, SGENod^−^-4, SGENod^−^-8	[7,31,57,61]
*sym39*	-	Nod^+/−^	P57	[7,62]
*sym40*	VII	Fix^−^	SGEFix^−^-1, SGEFix^−^-6	[49,58,63]
*sym42*	-	Fix^−^	RisFixV	[31,45]
*brz*	IV	Nod^−^	E107	[64]
*coch*	-	-	JI1824, JI3121, JI2165, JI2757, Wt11304, SGRcoch, SGEapm, FN3185/1325	[7,65,66,67,68,69,70,71]
*LykX*	-	-	JI 1357 (registered type line)	[72]
*k1*	I	Nod^−^/Nod^+/−^	Cameor 817, Cameor 885, Cameor 2265	[73]
*nof1*	-	Fix^−^	FN1	[74]
*nod1*	-	Nod^++^	Parvus	[5]
*nod2*	-	Nod^++^	Parvus	[5]
*nod3*	I	Nod^++^	nod3, P79, K10a, K11a, K12a, P79, RisFixC	[30,75,76,77]
*nod4*	V	Nod^++^	K301	[78,79]
*Nod5*	-	Nod^++^	Torsdag	[80]
*nod6*	VII	Nod^++^	K21a, K22a	[30,80]

* The dominant or recessive state of the allele determining the mutant phenotype is shown.
